# The Influence of Stress and Binge-Patterned Alcohol Drinking on Mouse Skeletal Muscle Protein Synthesis and Degradation Pathways

**DOI:** 10.3390/biom14050527

**Published:** 2024-04-28

**Authors:** Carter H Reed, Anna C. Tystahl, Hyeyoon Eo, Trevor J. Buhr, Ella E. Bauer, Ji Heun Lee, Peter J. Clark, Rudy J. Valentine

**Affiliations:** 1 Department of Biology, Grand View University, Des Moines, IA 50316, USA; creed@grandview.edu; 2 Department of Kinesiology, Iowa State University, Ames, IA 50011, USA; atystahl@iastate.edu (A.C.T.);; 3 Department of Food Science and Human Nutrition, Iowa State University, Ames, IA 50011, USA; 4 Department of Physical Therapy and Kinesiology, University of Massachusetts Lowell, Lowell, MA 01854, USA

**Keywords:** binge-patterned drinking, drinking in the dark, muscle protein synthesis, muscle protein degradation, autophagy

## Abstract

Adverse experiences (e.g., acute stress) and alcohol misuse can both impair skeletal muscle homeostasis, resulting in reduced protein synthesis and greater protein breakdown. Exposure to acute stress is a significant risk factor for engaging in alcohol misuse. However, little is known about how these factors together might further affect skeletal muscle health. To that end, this study investigated the effects of acute stress exposure followed by a period of binge-patterned alcohol drinking on signaling factors along mouse skeletal muscle protein synthesis (MPS) and degradation (MPD) pathways. Young adult male C57BL/6J mice participated in the Drinking in the Dark paradigm, where they received 2–4 h of access to 20% ethanol (alcohol group) or water (control group) for four days to establish baseline drinking levels. Three days later, half of the mice in each group were either exposed to a single episode of uncontrollable tail shocks (acute stress) or remained undisturbed in their home cages (no stress). Three days after stress exposure, mice received 4 h of access to 20% ethanol (alcohol) to model binge-patterned alcohol drinking or water for ten consecutive days. Immediately following the final episode of alcohol access, mouse gastrocnemius muscle was extracted to measure changes in relative protein levels along the Akt-mTOR MPS, as well as the ubiquitin-proteasome pathway (UPP) and autophagy MPD pathways via Western blotting. A single exposure to acute stress impaired Akt singling and reduced rates of MPS, independent of alcohol access. This observation was concurrent with a potent increase in heat shock protein seventy expression in the muscle of stressed mice. Alcohol drinking did not exacerbate stress-induced alterations in the MPS and MPD signaling pathways. Instead, changes in the MPS and MPD signaling factors due to alcohol access were primarily observed in non-stressed mice. Taken together, these data suggest that exposure to a stressor of sufficient intensity may cause prolonged disruptions to signaling factors that impact skeletal muscle health and function beyond what could be further induced by periods of alcohol misuse.

## 1. Introduction

One-in-four adults in the U.S. engage in alcohol binge drinking weekly, making it the predominant form of alcohol misuse in the United States [1]. While several risk factors can influence alcohol misuse, exposure to adverse experiences (e.g., psychological stress) is associated with an increased risk of regularly misusing alcohol [2,3,4]. Indeed, the risk of engaging in binge drinking can increase nearly three-fold following a traumatic event [5,6]. Both binge drinking and stress exposure are well-characterized as negatively affecting the cardiovascular and hepatic systems while also leading to hyperlipidemia and atherosclerosis [7,8,9,10]. However, a growing body of literature suggests that sufficiently prolonged and elevated stress responses and excessive alcohol ingestion can, independently, result in the development of skeletal muscle myopathy [10,11,12,13,14,15]. The loss of healthy skeletal muscle mass is associated with a reduced quality of life, particularly the reduced ability to perform daily activities [16]. Additionally, the loss of healthy skeletal muscle can lead to a dysregulation of metabolism, thereby increasing the risk of developing metabolic syndrome [17,18]. Therefore, understanding how stress exposure and alcohol misuse interact to affect protein synthesis pathways in skeletal muscle could inform strategies for muscle preservation in susceptible populations.

Skeletal muscle atrophy can occur when rates of muscle protein degradation (MPD) exceed synthesis (MPS) [19,20]. Several molecular signaling pathways influence the balance of MPS and MPD, including the Akt-mTOR pathway of MPS, the ubiquitin-proteasome pathway (UPP) of MPD, and skeletal muscle autophagy of MPD. Alcohol exposure can reduce MPS and increase MPD signaling pathways in rodent models; however, signaling alterations and effect sizes can be variable depending on the mode (e.g., intraperitoneal injection, gavage, voluntary, vapor chamber, etc.) and length of alcohol exposure in rodents [21,22,23,24]. A disruption in MPS and MPD signaling following alcohol exposure contributes to the atrophy of skeletal muscle [25,26] and loss of muscle function [27] following chronic periods of alcohol misuse. In addition, prolonged exposure to sufficiently elevated stress hormones can also induce a catabolic milieu in skeletal muscle that mirrors dysregulation in MPS and MPD signaling pathways observed with alcohol misuse [11,15]. As stress exposure is a significant risk factor for alcohol abuse, it is feasible that the two of these factors together could result in even more severe disturbances to skeletal muscle physiology and function. Thus, an investigation into how skeletal MPS and MPD are affected by a period of binge-patterned alcohol drinking following exposure to acute stress could offer insights into skeletal muscle myopathy within populations that may be particularly vulnerable, such as individuals with post-traumatic stress disorder (PTSD) who are at higher risk for alcohol abuse and dependence. However, how these two factors affect mammalian skeletal MPS and MPD remains unclear.

The purpose of this study was to investigate the influence of binge-patterned alcohol ingestion following exposure to an acute stressor on gastrocnemius MPS and MPD signaling pathways in adult male C57Bl/6J mice. Mice were exposed to a single episode of uncontrollable tail shocks (acute stress) as this model elicits physiological and behavioral responses in rodents that have been likened to a short-lived episode of PTSD [28,29,30,31,32,33,34,35]. Following exposure to acute stress, mice received daily intermittent access to alcohol in their home cages for ten consecutive days following the Drinking in the Dark (DID) paradigm, whereby mice reliably drink to blood alcohol levels exceeding 0.09 g/dL within two hours (i.e., National Institute on Alcohol Abuse and Alcoholism binge drinking standard) and display behaviors analogous to intoxication (e.g., anxiolysis, ataxia) [36,37,38]. Previous work from our lab has suggested that DID is a useful paradigm for understanding the detrimental effects of binge-like alcohol drinking on skeletal MPS and MPD [21]. Both signaling pathways of MPS and MPD, as well as rates of MPS, were assessed in the muscles of mice. The results of this study provide key insights into the interaction of prior exposure to an adverse experience and alcohol misuse on skeletal muscle physiology.

## 2. Materials and Methods

### 2.1. Animal Housing

Upon arrival, 9–10 weeks old male C57BL/6J mice (Jackson Labs, Bar Harbor, ME, USA) were individually housed. Mice were acclimated to a reversed 12:12 light-dark cycle for one week before beginning experimental procedures. During the study, mice had ad libitum access to standard chow (ENVIGO, Teklad 2014). Mice received ad libitum access to filtered water, except during the Drinking in the Dark procedure (described below). Research procedures were approved by the Iowa State University Institutional Animal Care and Use Committee (IACUC-23-046; approved on 3 October 2023).

### 2.2. Drinking in the Dark (DID) Paradigm

DID is a behavioral assay that models binge-patterned alcohol drinking using rodents. During the DID procedure, C57Bl/6J mice will reliably consume 20% alcohol to levels above the 0.09 g/dL blood alcohol content (BAC) within a 2 h period. A BAC above this level can induce behavioral outcomes similar to human intoxication, including ataxia and anxiolysis [36]. DID began three hours into the dark cycle, during the peak consummatory behavior of mice. Water bottles were replaced with graduated sipper tubes filled with 20% alcohol in filtered tap water. Mice received access to alcohol for 2 or 4 h each day of DID (see experimental design). Following alcohol access, sipper tubes were removed, and water bottles were returned to their cages until the next episode of DID. 

### 2.3. Uncontrollable Tail Shock Stress

The tail shock paradigm followed our previous publications [30,39,40]. The tail shock stress occurred 2–4 h into the dark cycle. Mice were restrained in individual plexiglass tubes with tails exposed. Electrodes were placed on the exposed tails, and 100 shocks averaging 1.25 mA (50 shocks at 1.0 mA and 50 shocks at 1.5 mA) were delivered. The duration of each shock was 5 s at variable 1 min intershock intervals. During tail shocks, control animals remained undisturbed in their home cages in a separate room. The uncontrollable tail shock paradigm was chosen because it is one of the most comprehensively investigated in stress physiology (for review, see [31]) and results in rodent physiological and behavioral outcomes that have been likened to PTSD [32,41]. Animals were monitored twice daily to ensure no prolonged distress over 48 h following stress exposure, as monitored by normal movement in cages, grooming, posture, and food consumption.

### 2.4. Experimental Design 

Mice participated in DID for four consecutive days to measure pre-stress exposure levels of alcohol ingestion. Following our previously published work [38], half of the mice received access to graduate sipper tubes containing alcohol in water during DID (alcohol), whereas the other half received access to water (controls). To acclimate to alcohol access, mice received access to sipper tubes with either alcohol or water for 2 h per day during the first three days of DID. On the fourth day, mice were allowed 4 h of access. Seventy-two hours after day four of DID, half of the mice were exposed to the tail shock protocol (acute stress, described above) or were left undisturbed in home cages (no stress). Mice were left undisturbed post-stress exposure for another 72 h before an additional ten consecutive days of DID, where 20% alcohol (i.e., alcohol group) or water (i.e., water group) access was permitted for 4 h per day. The pre- and post-72 h period without alcohol access was completed to reduce the possibility of mice associating access to alcohol directly with exposure to stress. Thus, the experimental design consisted of four groups: No-Stress/Water (*n* = 9), No-Stress/Alcohol (*n* = 9), Stress/Water (*n* = 9), and Stress/Alcohol (*n* = 9). 

Mice were injected with puromycin dissolved in saline at a concentration of 0.04 μmol/g of body weight 3.5 h into the final episode of DID. Puromycin delivery in small amounts allows its incorporation into newly synthesized peptides, measuring protein synthesis in muscle [42,43]. The concentration of puromycin and time of injection were chosen based on previously published protocols to allow for puromycin incorporation into newly synthesized peptides, and puromycin has also been used to assess the effects of alcohol on muscle protein synthesis [44]. 

**Figure 1:**
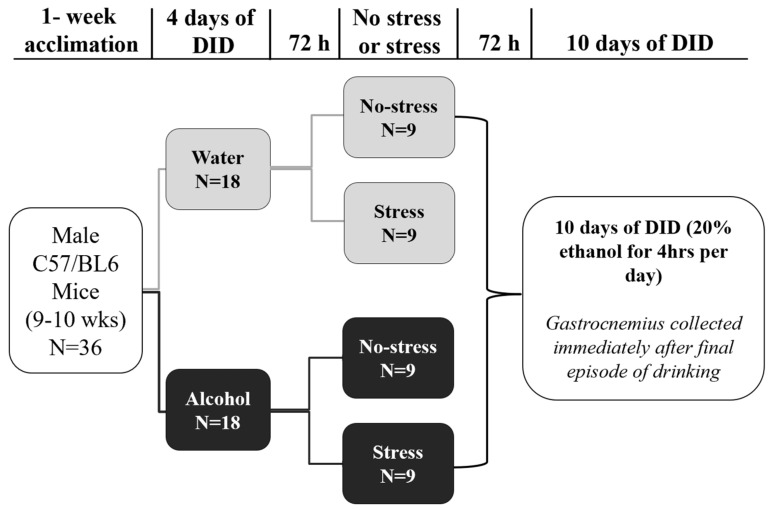
The experimental design. Male mice participated in “Drinking in the Dark” (DID), where they received access to 20% alcohol or water (controls) two hours daily over three days and four hours on the fourth day. Seventy-two hours later, mice were exposed to a single episode of acute stress or left undisturbed in home cages (no stress). Seventy-two hours later, mice participated in daily bouts of DID, where they received 4 h of access to 20% alcohol or water for 10 consecutive days.

Thirty minutes after puromycin injection during DID, mice were euthanized via rapid decapitation without anesthesia. The gastrocnemius muscle was quickly excised and placed in a microtube and flash-frozen in liquid nitrogen. Samples were stored at −80 °C until Western blot analysis. A visual representation of the current experimental design is depicted in Figure 1. 

### 2.5. Western Blot Analysis

Changes in relative protein expression were analyzed using Western blot analysis per our previously published protocols [21,30,45]. Roughly 50 mg of gastrocnemius was homogenized in 10× volume of ice-cold RIPA buffer (Thermo Fisher Scientific, Waltham, MA, USA) in combination with protease inhibitor and phosphorylase inhibitor (Thermo Fisher Scientific, Waltham, MA, USA and Sigma-Aldrich, Burlington, MA, USA, respectively). After homogenization, samples were centrifuged at 18,000× *g* at 4 °C for 10 min, and protein was extracted and then quantified using a Pierce BCA Protein Assay kit (Thermo Fisher Scientific, Waltham, MA, USA). Equal amounts of protein (25 µg) were loaded into 4–15% or 4–20% Criterion TGX Stain-Free gels, and proteins were separated using electrophoresis as were molecular weight markers to allow for accurate processing for proteins of interest. Following separation, gels were activated under U.V. Then, proteins were transferred to polyvinylidene difluoride (PVDF) membranes (Millipore Sigma, Burlington, MA, USA). After transfer, U.V. transillumination images were captured for total lane protein normalization. Next, membranes were blocked at room temperature for one hour in 5% non-fat dry milk, washed in TBST, then incubated in primary antibody at 1:1000–1:2000 dilution overnight at 4 °C. The next day, membranes were washed and incubated in HRP-conjugated secondary antibody at a 1:5000 dilution at room temperature (anti-rabbit IgG #7074S or anti-mouse IgG #7076S; Cell Signaling) for 1 h, washed several more times, and incubated in Supersignal West Pico Plus Chemiluminescent Substrate or Supersignal ELISA Femto Maximum Sensitivity Substrate (Thermo Fisher Scientific, Waltham, MA, USA) and imaged using the ChemiDoc XRS Imaging System (Bio-Rad, Hercules, CA, USA). 

Protein densitometry was performed with Image Lab 6.0.1 software from Bio-Rad. Both high concentrations of alcohol and repeated episodes of stress exposure can affect signaling proteins along the Akt-mTOR pathway [23,25,46]. Thus, the following markers were analyzed in the gastrocnemius, phosphorylated-Akt^S473^ (#9271), Akt (#9272), phosphorylated-mTOR^S2448^ (#2971), mTOR (#2983), phosphorylated-4E-BP1^T37/46^ (4E-BP1, #2855), 4E-BP1 (#9644), phosphorylated-p70S6K^T389^ (#9234), and p70S6K (#2708); all markers were purchased from Cell Signaling Technology (Danvers, MA, USA). 

High volumes of alcohol can also affect autophagy signaling pathways in skeletal muscle [47]. Moreover, the influence of stress exposure on this pathway of MPD has yet to be thoroughly examined. Therefore, we analyzed vital signaling proteins involved in skeletal muscle autophagy: phosphorylated-ULK1^S757^ (#6888S) and LC3 (#12741), which were purchased from Cell Signaling Technology. We also analyzed ULK1 (#sc-390906), which was purchased from Santa Cruz Biotechnology (Dallas, TX, USA) and p62 (ab109012) was purchased from Abcam (Boston, MA, USA). 

Alcohol misuse and chronic stress exposure can also increase myostatin, an activator of ubiquitin-proteasome-mediated protein degradation [15,48]. Therefore, we assessed changes in relative protein expression of the precursor myostatin protein (#sc-134345; Santa Cruz Biotechnology), commonly used to determine myostatin protein expression [49,50]. Ubiquitin (#20326; Cell Signaling Technology), as well as the relative protein expression of the E3 ligases MAFbx (#sc-166806; Santa Cruz Biotechnology) and MuRF-1 (#sc-398608; Santa Cruz Biotechnology), were also assessed. The effects that binge-patterned drinking or acute stress exposure may have on cellular stress remain to be elucidated. However, through disruption in the signaling of the previously mentioned pathways, a disruption in cellular homeostasis may occur. Therefore, HSP70 (#sc-24; Santa Cruz Biotechnology) was analyzed as a marker of cellular stress. Finally, relative protein expression of puromycin (MABE342; Millipore Sigma, Burlington, MA, USA) incorporation was used to assess rates of muscle protein synthesis. Original western blots can be found in Supplementary Materials.

### 2.6. Statistical Analysis

Statistical analyses were performed using GraphPad Prism 9 software (GraphPad Software Inc., San Diego, CA, USA). Data were presented as mean ± standard error of the mean (SEM). A two-way analysis of variance (ANOVA) was completed with alcohol (i.e., alcohol vs. water) and stress (i.e., stress vs. no stress) as factors for MPS and MPD signaling protein expression, percentage body weight change, and average daily food intake. Post hoc *t*-tests with Tukey corrections for multiple comparisons were performed on significant interactions between alcohol access and stress. Repeated measures ANOVA (RMANOVA) was completed on daily drinking volumes during DID, with alcohol and stress as between-subject factors and day as the within-subject factor. Post hoc analyses were performed using Tukey’s correction for multiple comparisons of interactions that reached statistical significance. Significance was set at *p* < 0.05 for all analyses. Statistically nonsignificant trends are reported up to *p* < 0.1. 

## 3. Results

### 3.1. Body Mass, Food Intake, and DID

Body mass and alcohol ingestion are presented in Table 1. Mice gained weight throughout the study (*p* = 0.002; Table 1). No main effect of stress (*p* = 0.352) or alcohol (*p* = 0.612) was observed on the percentage of body weight change. No main effects of stress (*p* = 0.462) or alcohol (*p* = 0.462) were observed for daily food consumption relative to body weight across the duration of the study.

Mice with access to alcohol drank an average of 6.9 g/kg of ethanol per day during four hours of voluntary alcohol consumption, which equates to a blood alcohol level exceeding 1.0 mg/mL established in our laboratory and others [37,38]. A significant main effect of the day (*p* < 0.001) was observed, whereby daily drinking volumes varied across days. However, relative alcohol consumption between days was not significantly different (*p* = 0.191). Additionally, no interaction between stress and day (*p* = 0.372) on fluid consumption relative to body weight was observed, suggesting stress did not influence fluid ingestion. Throughout the study, mice with access to alcohol consumed more fluid per kilogram of body weight than those with access to water (*p* < 0.001). No difference in grams of alcohol ingested per kilogram of body weight was observed between non-stressed and stressed animals throughout the study (*p* = 0.218). 

### 3.2. Akt-mTOR Signaling

**Figure 2:**
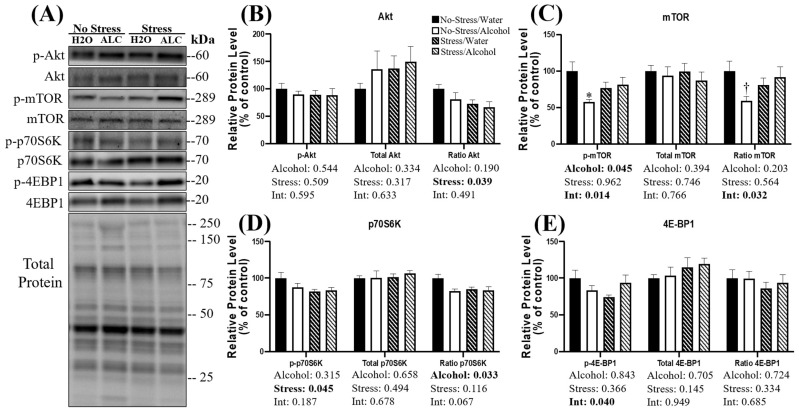
Changes in relative protein expression of the Akt-mTOR signaling pathway following acute stress and alcohol access. Representative blots (**A**) and quantification (**B**–**E**) are presented for several key proteins in the Akt-mTOR pathway. Data are presented as mean ± SEM (*n* = 9/group). Two-way ANOVA was used to compare both the main effects and an interaction between stress and alcohol (listed below the *x*-axis). Tukey’s post hoc analysis was used for multiple comparisons, and statistical significance was set at *p* < 0.05. * Denotes significantly decreased compared to no-stress/water group (*p* < 0.05); ^†^ Denotes a non-statistically significant trend compared to no-stress/water group (*p* < 0.10).

The canonical Akt-mTOR pathway is a crucial regulator of muscle protein synthesis [51], and phosphorylation of protein kinase B (i.e., Akt) leads to increased protein synthesis [52]. No effect of stress, alcohol, or a combination of factors was observed on the phosphorylated Akt or total Akt (*p* > 0.317; Figure 2B). However, a main effect of stress (*p* = 0.039) was observed on the ratio of p-Akt/Akt, whereby mice exposed to stress had lower Akt phosphorylation than non-stressed mice. However, no main effect of alcohol (*p* = 0.190) or interaction between stress and alcohol (*p* = 0.491) was observed. 

The activation of Akt results in phosphorylation of the mammalian target of rapamycin (mTOR) [53]. A main effect of alcohol was observed on the phosphorylated mTOR, whereby alcohol access significantly decreased mTOR phosphorylation (*p* = 0.045; Figure 2C). Furthermore, a significant interaction between alcohol and stress was observed (*p* = 0.014), and post hoc analysis revealed that in non-stressed animals, alcohol access significantly decreased the phosphorylation of mTOR (*p* = 0.012). No main effect of stress was observed on mTOR phosphorylation (*p* = 0.962). Similarly, there was no main effect of alcohol (*p* = 0.394), stress (*p* = 0.746), or interaction between factors (*p* = 0.766) observed on the relative protein expression of total mTOR. No main effect of stress (*p* = 0.564) or alcohol (*p* = 0.203) was observed on the ratio of p-mTOR/mTOR; however, a significant interaction between stress and alcohol (*p* = 0.032) was observed, whereby the phosphorylation status of mTOR was highest in the control animals. Post hoc analysis revealed a statistically non-significant trend towards a reduced phosphorylation status of mTOR in non-stressed mice with alcohol access compared to non-stressed mice with water access during DID (*p* = 0.079), suggesting alcohol access alone may reduce mTOR phosphorylation. 

P70 S6 kinase (p70S6K) is a substrate phosphorylated by mTOR, leading to increased protein synthesis. A main effect of stress was observed, whereby mice exposed to stress had lower p70S6K phosphorylation (*p* = 0.045; Figure 2D). However, neither a main effect of alcohol (*p* = 0.315) nor an interaction between alcohol and stress was observed on p70S6K phosphorylation (*p* = 0.187). No main effect of alcohol (*p* = 0.658), stress (*p* = 0.494), or interaction between factors (*p* = 0.678) was observed on the relative expression of total p70S6K. A main effect of alcohol was observed on the ratio of p-p70S6K/p70S6K (*p* = 0.033), whereby alcohol access reduced phosphorylation compared to water access during DID. No main effect of stress (*p* = 0.116) on p-p70S6K/p70S6K was observed; however, a statistically non-significant trend toward an interaction between stress and alcohol (*p* = 0.067) was observed. 

Finally, eukaryotic translation initiation factor 4E-binding protein 1 (4E-BP1) is another substrate phosphorylated by mTOR. A significant interaction between alcohol and acute stress was observed on 4E-BP1 phosphorylation (*p* = 0.040; Figure 2E), and mice exposed to stress that consumed water during DID appeared to have the lowest levels of 4E-BP1 phosphorylation. No main effect of alcohol (*p* = 0.843) or main effect of stress (*p* = 0.366) was observed. Furthermore, neither a main effect of alcohol (*p* = 0.705), stress (*p* = 0.145), nor interaction between factors (0.949) was observed on the relative protein expression of 4E-BP1. The ratio of p-4E-BP1/4E-BP1 was not affected by a main effect of stress (*p* = 0.334), alcohol (0.724), or interaction between these factors (*p* = 0.685). 

### 3.3. Ubiquitin Proteasome Pathway Signaling

**Figure 3:**
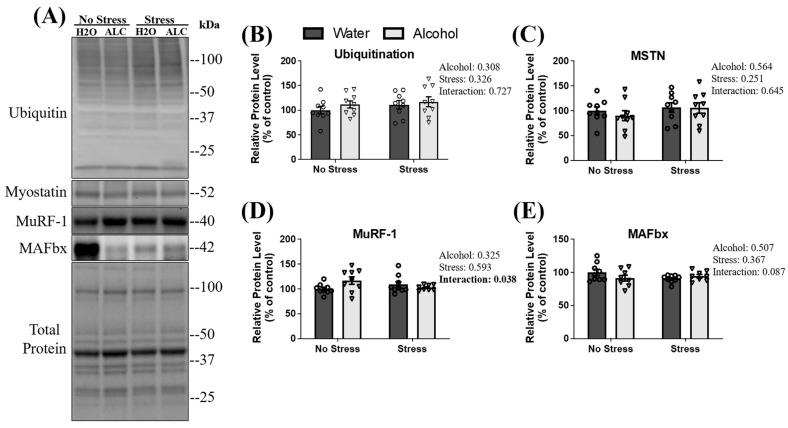
Changes in the ubiquitin-proteasome pathway and myostatin following acute stress and alcohol access. Both representative blots (**A**) and relative protein expression (**B**–**E**) are presented. Data are presented as mean ± SEM (*n* = 9/group). A two-way ANOVA was used to compare the individual effects of stress exposure and binge-patterned drinking, as well as to compare and interaction between factors (reported next to each figure).

The ubiquitin-proteasome pathway (UPP) is a potent regulator of muscle mass, and overactivation leads to the atrophy of skeletal muscle [54,55]. Briefly, in UPP-mediated protein degradation, proteins destined for degradation are poly-ubiquitinated, leading to eventual degradation by the proteasome. No main effect of stress (*p* = 0.326), alcohol (*p* = 0.308), or an interaction between factors was observed on the poly-ubiquitination of proteins (*p* = 0.727; Figure 3B). Myostatin is a negative regulator of skeletal muscle mass that can influence UPP signaling in skeletal muscle [56]. No main effect of stress (*p* = 0.251), alcohol (*p* = 0.564), or an interaction between these factors was observed (*p* = 0.645; Figure 3C) for the relative protein expression of myostatin. Two components of UPP signaling in skeletal muscle are the E3 ligases muscle ring finger-1 (MuRF-1) and muscle atrophy f-box (MAFbx) [57,58]. A significant interaction between stress and alcohol (*p* = 0.038) on MuRF-1 protein expression was observed, whereby relative protein levels of MuRF-1 appeared lowest in non-stressed mice with access to water compared to the other groups. However, post hoc analysis failed to reveal group differences in MuRF-1 expression. Moreover, neither stress (*p* = 0.593) nor alcohol (*p* = 0.325) affected MuRF-1 expression (Figure 3D). There was no main effect of stress exposure (*p* = 0.367) or alcohol access (*p* = 0.507) on the relative protein expression of MAFbx. However, a statistically non-significant trend towards an interaction between these factors (*p* = 0.087; Figure 3E) was observed. 

### 3.4. Autophagy Pathway Related Signaling

**Figure 4:**
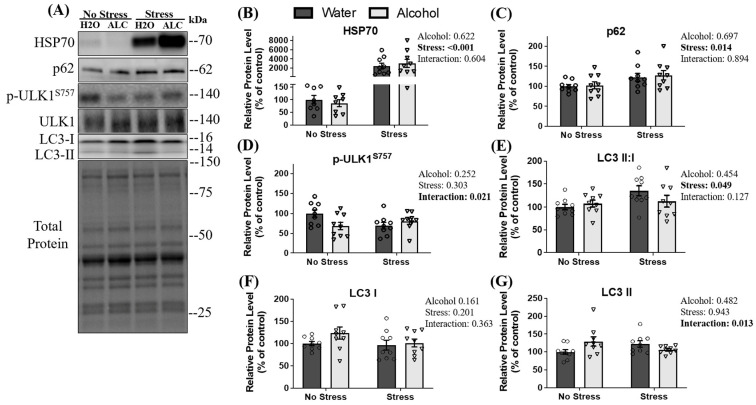
The effects of stress exposure and binge-patterned alcohol drinking on autophagy signaling and HSP70. Representative blots (**A**) and relative protein expression (**B**–**G**) are presented. Data are presented as mean ± SEM (*n* = 9/group). To compare the effects of acute stress, alcohol consumption, and an interaction between factors, a two-way ANOVA was used. Statistical significance was set at *p* < 0.05 (reported next to each figure).

Heat shock protein 70 (HSP70) was assessed as it is rapidly expressed to maintain proteostasis and cell integrity [59] as a response to stimuli such as heat stress, increased ROS activity, or injury to skeletal muscle. HSP70 additionally plays an important role in chaperone-assisted UPP degradation and chaperone-assisted autophagy [60]. A main effect of stress (*p* < 0.001; Figure 4B) was observed, whereby tail shock exposure increased the relative expression of HSP70 by almost 3000% compared to animals not exposed to stress. However, no main effect of alcohol (*p* = 0.622) or interaction between stress and alcohol (*p* = 0.604) was observed for HSP70. Another protein degradation pathway is autophagy, which clears damaged proteins to maintain cellular health [61]. Therefore, signaling proteins involved in autophagy were assessed. First, sequestosome 1, p62, was analyzed as it is involved in both UPP and autophagy-related protein degradation [62]. A main effect of stress (*p* = 0.014; Figure 4C) was observed, whereby stressed mice had a higher relative protein expression of p62 compared to non-stressed mice. No main effect of alcohol (*p* = 0.679) or interaction between factors (*p* = 0.894) was observed for p62. 

Formation of the autophagosome is initiated by unc-51-like autophagy activating kinase 1 (ULK1), and the activation of ULK1 is blunted following phosphorylation at serine 757 by mTOR, preventing the induction of autophagy [63]. A significant interaction between stress and alcohol (*p* = 0.021) was observed on ULK1 phosphorylation at serine 757 (normalized to total ULK1), whereby control mice appeared to have the highest relative levels of phosphorylation at serine 757 and alcohol alone, stress alone, and the combination of factors appeared to reduce inhibition of ULK1. Further, post hoc analysis revealed that non-stressed mice with water access had a statistically non-significant trend towards more p-ULK1^S757^ than stressed mice with water access (*p* = 0.086) and non-stressed mice with alcohol access (*p* = 0.072), suggesting stress alone may reduce the phosphorylation of ULK1^S757^. No main effect of stress (*p* = 0.303) or alcohol (*p* = 0.252) on the phosphorylation of ULK1 at serine 757 was observed (Figure 4D). 

As an estimate of autophagosome formation, the ratio of microtubule-associated protein 1A/1B-light chain 3 (LC3) form II: I was assessed [64]. A main effect of stress was observed on the ratio of LC3 II: I ratio (*p* = 0.049; Figure 4E), whereby mice exposed to acute stress had a significantly increased ratio of LC3 II:I. No main effect of alcohol (*p* = 0.454) or interaction between alcohol and stress (*p* = 0.127) was observed in this ratio. 

### 3.5. Muscle Protein Synthesis 

**Figure 5:**
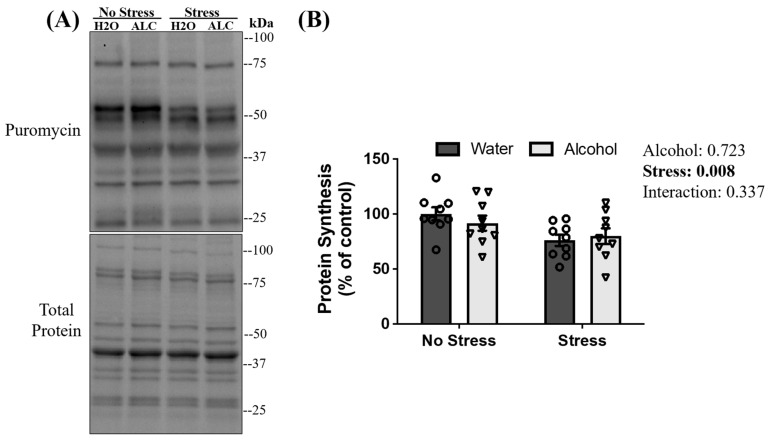
Puromycin incorporation to peptide chains following acute stress and alcohol access. Representative blots (**A**) and quantification of puromycin levels (**B**) are presented. Data are presented as mean ± SEM (*n* = 9/group). Two-way ANOVA assessed the significant main effects and interactions between alcohol and stress (reported next to the figure).

Prolonged decreases in MPS can lead to skeletal muscle atrophy [65]. Puromycin injections and incorporation into skeletal muscle are used to measure protein synthesis in skeletal muscle [66,67]. A main effect of stress exposure (*p* = 0.008) was observed, whereby diminished rates of puromycin incorporation into skeletal muscle were observed 13 days after the episode of tail shocks in mice exposed to acute stress compared to non-stressed mice. However, there was no main effect of alcohol (*p* = 0.713) or interaction between stress and alcohol (*p* = 0.337; Figure 5B) on puromycin incorporation.

## 4. Discussion

Exposure to adverse experiences can precipitate periods of alcohol misuse [2,3,4]. The impact of adverse experiences coupled with binge-patterned alcohol drinking on signaling pathways involved in muscle proteostasis remains unexplored despite evidence supporting that each of these factors independently is detrimental to skeletal muscle health. The results of the study revealed a few noteworthy findings. A single episode of acute stress caused a prolonged depression in rates of MPS. Indeed, a lowering of puromycin incorporation into peptide chains in mouse gastrocnemius muscle was detected on day thirteen after the initial stress exposure. This finding was consistent with an acute stress-related reduction in phosphorylation of signaling molecules along the Akt-mTOR pathway, co-occurring with the modulation of autophagy signaling factors. Interestingly, while daily intermittent alcohol access did not influence overall rates of puromycin incorporation in mouse gastrocnemius muscle, Akt-mTOR signaling cascades were disrupted in a manner that could become detrimental to MPS [24,68]. Finally, a few interactions between stress exposure and alcohol drinking were observed along the Akt-mTOR and autophagy pathways. These interactions overall seem to reflect the detrimental effects of alcohol on the MPS and MPD signaling pathways in mice not exposed to stress, as stress exposure may have lowered MPS and elevated MPD signaling beyond what could be further detected by binge-like alcohol ingestion. Taken together, the observations from our study suggest that adverse experiences and binge-patterned alcohol drinking may both be detrimental to MPS and MPD signaling pathways in the mouse gastrocnemius muscle. However, exposure to an adverse event of sufficient intensity may persistently lower MPS and augment MPD signaling beyond what can be further detected by a period of binge-patterned alcohol drinking. 

Repeated exposure to restraint stress can result in muscle atrophy [11,15,46,69], yet whether rates of MPS are sensitive to stress exposure versus repeated bouts of immobility is less clear. Our data, in which MPS was suppressed nearly two weeks after stress, suggest that stress per se may contribute to a possible long-term disruption in muscle protein balance, although the immobility may further promote muscle atrophy, as observed in chronic restraint models [11,46], that was not observed here. Further, the synthesis of new proteins in skeletal muscle is regulated by the availability of amino acids consumed in the diet. Stress-reduced food intake may also be associated with skeletal muscle atrophy, which has been observed in at least one investigation [15]. However, in the current study, diminished rates of MPS in stressed animals appear to be independent of nutritional status, as no difference in food intake between stressed and non-stressed animals was observed. Therefore, impairments within skeletal MPS signaling pathways regulating proteostasis can be attributed more closely to stress and not immobility or alterations in food intake, which are factors to consider in previous reports. 

In the current study, the phosphorylation of Akt, an integral signaling protein involved in the stimulation of MPS, was also downregulated nearly two weeks after stress exposure. Furthermore, stress exposure also decreased phosphorylated p70S6K, a downstream protein in the Akt-mTOR pathway. These findings support other studies suggesting repeated episodes of daily stressors can reduce the phosphorylation of Akt and its related signaling in skeletal muscle [11,46]. Notably, reduced activity of Akt alone is often sufficient to induce atrophy. For example, activation of mTOR and downstream targets does not rescue atrophy induced by the knockout of Akt in mice [70]. Akt, therefore, may play a prominent mTOR-independent role in controlling MPS. Thus, it is feasible that reductions in Akt activity may be sufficient to reduce rates of MPS and may not require modulations of protein phosphorylation status downstream. It is worth noting that studies using chronic restraint stress, 8 h a day for five days a week, have reported increased phosphorylation of several proteins along the Akt-mTOR pathway [11]. Thus, taken together, our results suggest that exposure to a single episode of acute stress results in a prolonged deficit of MPS by most prominently disrupting Akt. However, the downstream target p70S6K may also be negatively regulated by stress exposure.

Myostatin is a negative regulator of muscle mass proposed to be an essential signaling hub involved in chronic stress-induced atrophy [15,46], whereby its absence can prevent muscle atrophy [15]. Mechanistically, myostatin can reduce Akt-mTOR signaling while increasing the transcription of the UPP ligases MuRF-1 and MAFbx [71]. Chronic restraint stress has been shown to induce the expression of myostatin, which could also contribute to muscle atrophy [15,46]. However, in the current study, the relative protein expression of myostatin was unchanged at two weeks following stress exposure, yet the Akt-mTOR pathway and MPS synthesis were still dampened. Increased myostatin expression is typically associated with an increase in glucocorticoids [72,73], and it has been well established that in the current model of stress exposure, an increase in corticosterone is observed in the days following stress but returns to pre-stress levels when measured 96 h later [74]. Thus, at the time point muscle was collected, no changes in corticosterone were necessarily expected, and the lack of changes in myostatin and related UPP signaling due to stress exposure aligns with that expectation. It is also plausible that restricted locomotor activity during chronic restraint stress may also be necessary for myostatin-induced reductions in MPS [75]. The current study employed a single episode of acute stress exposure, which does not cause long-term or repeated restrictions in movement like chronic restraint stress. The lack of observed changes to myostatin levels, along with reductions in Akt following stress exposure, may further tease apart biological mechanisms behind stress-related deficits in MPS compared to potentially confounding methodological factors with other stress models.

Data from the current study suggest that exposure to an adverse experience may also cause long-term disruptions in MPD autophagy processes, as integral autophagy signaling proteins were modulated following stress exposure. The proper function of skeletal muscle and maintenance of myofiber integrity relies on appropriate autophagy signaling. Indeed, when autophagy signaling is inhibited, profound reductions in muscle mass can occur, resulting in impaired force production [76,77]. The expression of p62 and the LC3 II: I ratio was increased following stress exposure. P62 is important in linking cargo destined for degradation to the autophagosome. When autophagy becomes compromised, p62 can accumulate because autophagic processes cannot readily clear it [76]. Similarly, the comparative increase in LC3 II also suggests an increase in autophagosome accumulation as the normal autophagic signaling cascade becomes dysregulated [78]. Moreover, oxidative stress can disrupt autophagy, leading to an accumulation of p62 and LC3-II, while damaged cellular components are not efficiently removed [79]. Consistent with these observations, ULK1, a complex that becomes activated in a proteotoxic environment and influences phagophore formation, increases activity in situations where protein aggregates accumulate and are not efficiently cleared by autophagy [80]. The dephosphorylation of ULK1 at serine 757 can contribute to activating the ULK1 complex under such conditions. Interestingly, data from the current study found more dephosphorylation of serine 757 in ULK1 in the stressed compared to the non-stressed mice, which could be consistent with more ULK1 activation due to damaged cellular proteins accumulating in cellular space, yet this was only observed in mice consuming water throughout the study. Therefore, a possibility remains that damaged cellular components in muscles following stress may not be effectively cleared due to disruptions in autophagic processes. The effects of acute stressors on autophagic flux and overall muscle health should be a topic of further investigation, as it could provide valuable information for elucidating stress-induced myopathy in vulnerable populations. 

Further evidence that adverse events can persistently increase myocellular stress was also observed by a nearly 30-fold increase in relative protein expression of HSP70. HSP70 is a chaperone protein reported to rapidly increase in response to various cellular challenges, such as heat and oxidative stress, to maintain homeostasis and prevent cell death [59,60,81]. Furthermore, in myofibers, the induction of HSP70 following exposure to a stressor is critical for avoiding myopathy. Thus, a relatively persistent increase in skeletal muscle cellular stress, marked by a rise in HSP70, may accompany additional signaling dysregulation from exposure to an adverse experience of sufficient intensity. The persistent effects of adverse experiences on skeletal muscle health remain underexplored, but characterizing these patterns of signaling perturbations may be necessary for developing therapeutic approaches to combat long-term muscle dysfunction following stress-related conditions such as PTSD. 

In contrast to stress exposure, ten days of binge-patterned alcohol ingestion did not influence puromycin incorporation in mouse gastrocnemius muscle. However, notable disruptions were observed along the Akt-mTOR pathway that could be consistent with the impaired MPS reported by other studies [46,69]. Alcohol access decreased the phosphorylation of p70S6K and p-mTOR levels. A reduction in the phosphorylation of both proteins has been reported following rodent models of chronic alcohol drinking [82,83]. The DID paradigm, which models binge-patterned alcohol consumption, the most common form of alcohol misuse, is consistent with the outcomes of chronic alcohol-only ingestion in rodents. 

When MPS signaling is impaired, compensatory activation of several signaling pathways increases transcription of ribosomal RNA, thus allowing MPS to continue in an mTOR-independent manner [84,85]. In fact, the reduction in the phosphorylation of several proteins along the Akt-mTOR pathway with a simultaneous compensatory increase in activation of a separate path that can augment MPS independent of mTOR has been observed following chronic alcohol ingestion in skeletal muscle [86]. Another feasible explanation for unchanged MPS due to alcohol consumption may be simply due to the current study design. Chronic models of alcohol misuse, where rats have continuous access to sweetened alcohol for as long as 26 weeks, observed reductions in MPS [25]. Furthermore, reports observing acute depressions in MPS following intoxication typically expose animals to alcohol (via gavage or intraperitoneal injection) in amounts that can result in a BAC exceeding 0.3 g/dL throughout a 1.5 h period. These BACs greatly exceed those reported by voluntary alcohol ingestion during DID in our study, which reliably achieved BACs over 0.1 g/dL following 2–4 h of alcohol access [37,38], more consistent with typical human patterns of alcohol misuse. Therefore, timing, mode, and length of alcohol exposure may be important considerations for capturing early or more robust reductions in MPS.

The combination of stress exposure and alcohol access resulted in few significant interactions on signaling molecules along MPS, MPD, and UPP pathways. This is surprising because prolonged exposure to sufficiently elevated stress hormones can also induce a catabolic milieu in skeletal muscle that mirrors dysregulation in MPS and MPD signaling pathways observed with alcohol misuse [11,15]. However, it is worth noting most of the stress and alcohol interactions reported in this study suggest that alcohol access in non-stressed mice altered relative MPS, MPD, or UPP signaling protein expression to levels comparable to stressed mice with and without alcohol access. For instance, significant and trending interactions observed with p-ULK^S757^, p-mTOR, and P70S6K display a reduction in these factors in non-stressed alcohol-drinking mice to levels comparable to stressed mice. These data suggest that acute stress exposure may alter proteostatic signaling molecules to levels that could not be further affected by binge-patterned drinking. It is, instead, possible that alcohol and stress interactions could be more readily observable following exposure to milder forms of stress or after even greater volumes of alcohol ingestion than reported in the current study.

While dysfunction along MPS and MPD signaling pathways was detected by stress and alcohol, which reduced MPS detected even two weeks after stress, a limitation was that lean body mass or muscle mass measurements were not obtained for this study and could be topics for future studies. However, it should be noted that a loss of muscle mass and function has been reported in individuals who have experienced psychological trauma (e.g., PTSD or ACEs) [87,88,89], which could be due to several factors secondary to the adverse experience, including changes to diet [90,91] or physical activity status [92,93], that has not been fully uncovered. The current findings suggest muscle myopathies following exposure to adverse experiences may be related to long-term physiological deficiencies along MPS and MPD pathways following adverse experiences and not stress-related secondary outcomes like dietary deficiencies or changes to physical activity status. Thus, these data provide evidence for the direct influences of acute stress and binge-patterned alcohol ingestion in modifying MPS and MPD pathways, providing possible important clinical context for understanding muscle myopathies reported following these conditions.

## 5. Conclusions

In conclusion, evidence from the current investigation demonstrates that a single exposure to acute stress may cause a lasting reduction in MPS and related signaling molecules while also disrupting biological substrates involved in autophagy. These data may indicate a relatively persistent state of cellular stress following exposure to adverse experiences. Interestingly, no evidence was found supporting the further impairment of muscle homeostasis in stressed animals by a period of binge-patterned alcohol ingestion, despite alcohol ingestion disrupting mTOR signaling in non-stressed animals. Taken together, these data suggest that exposure to a stressor of sufficient intensity may cause lasting disruptions to muscle homeostasis, but disruptions were not exacerbated by binge drinking. These data further our understanding of the detrimental effects of adverse experiences on mammalian skeletal myopathy, which remains a relatively less investigated tissue source compared to other organs like the liver, brain, and heart. These data could also have important implications for muscle health and function for individuals exposed to psychological traumas, such as those affected by PTSD.

## Figures and Tables

**Table 1:** Changes in body weight, food intake, drinking volumes, and ethanol ingestion.

	No Stress Water	No Stress Alcohol	Stress Water	Stress Alcohol
Initial body weight (g)	25.9 ± 0.3	25.1 ± 0.6	25.1 ± 0.6	25.3 ± 0.6
Final body weight (g)	26.6 ± 0.4	25.5 ± 0.6	26.1 ± 0.7	25.8 ± 0.4
Food intake (g) (post-stress)	4.0 ± 0.2	3.8 ± 0.1	3.9 ± 0.2	3.6 ± 0.1
2 h Drinking Volume (mL)	0.2 ± 0.03	0.5 ± 0.04 ^#^	0.2 ± 0.03	0.5 ± 0.03 ^#^
2 h Ethanol (g/kg)	N/A	3.3 ± 0.3	N/A	3.2 ± 0.2
4 h Drinking Volume (mL) (pre-stress)	0.3 ± 0.1	1.1 ± 0.1 ^#^	0.2 ± 0.1	1.0 ± 0.1 ^#^
4 h Drinking Volume (mL) (post-stress)	0.4 ± 0.1	1.3 ± 0.1 ^#^	0.4 ± 0.2	1.0 ± 0.1 ^#^
4 h Ethanol (g/kg) (post-stress)	N/A	8.2 ± 0.7	N/A	6.5 ± 0.6

Data are presented as mean ± SEM (*n* = 9/group). ^#^ significantly increased compared to water-drinking mice (*p* < 0.001). N/A: mice assigned to water group did not consume ethanol.

## Data Availability

Data available on request from the corresponding author.

## Supplementary Materials

The following supporting information can be downloaded at: https://www.mdpi.com/article/10.3390/biom14050527/s1.
