# Supplementary material for: The Influence of Stress and Binge-Patterned Alcohol Drinking on Mouse Skeletal Muscle Protein Synthesis and Degradation Pathways
Source: Biomolecules. 2024 Apr 28;14(5):527. doi: 10.3390/biom14050527 (PMC11118922; doi:10.3390/biom14050527)
Supplement: Supplementary file 1 [file biomolecules-14-00527-s001.zip › biomolecules-2893032-supplementary.pdf]

Fig 2

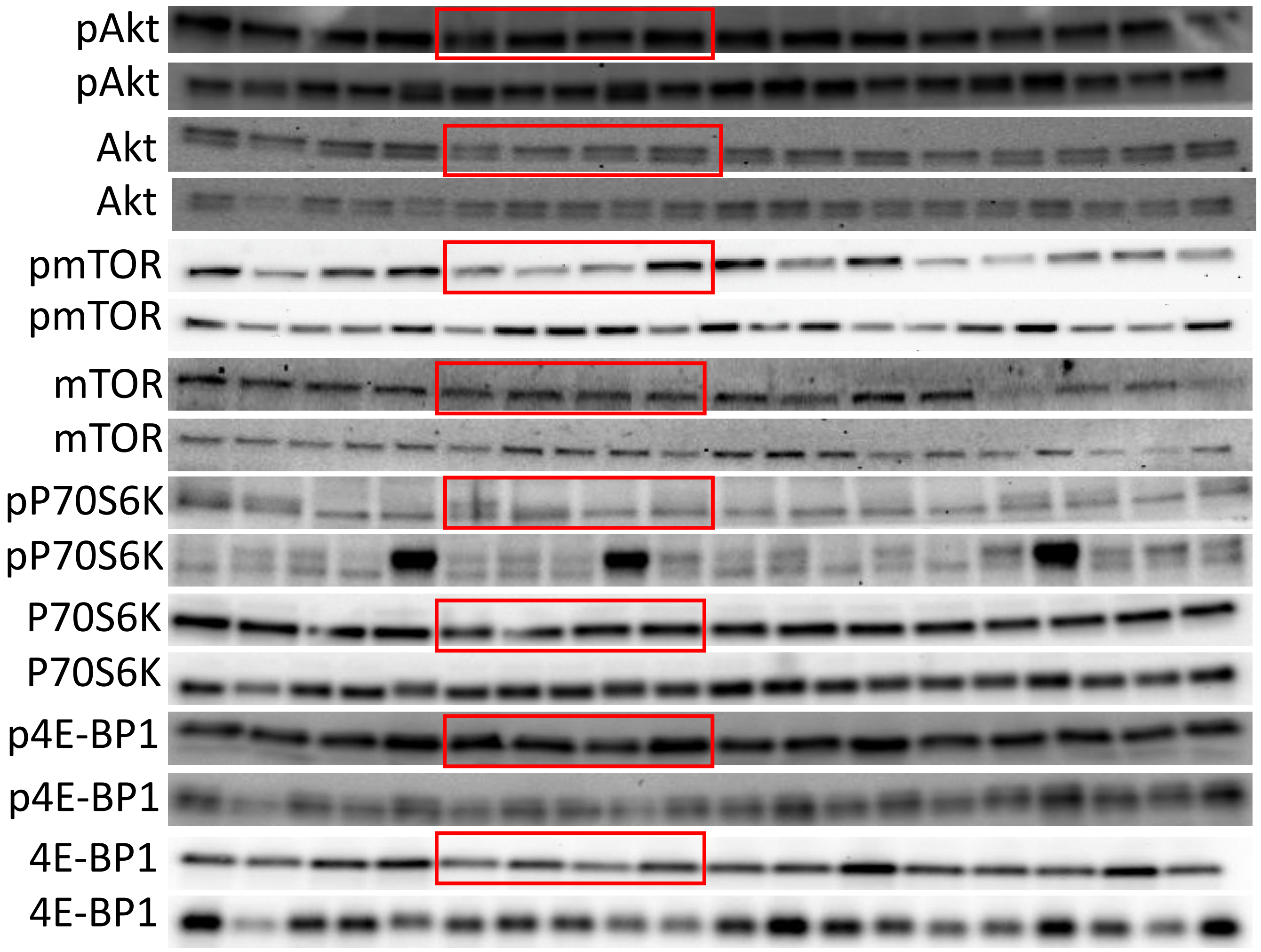

From left to right, blots are in repeats of No-stress water (control), no-stress alcohol, stress water, and stress alcohol.

Fig 3

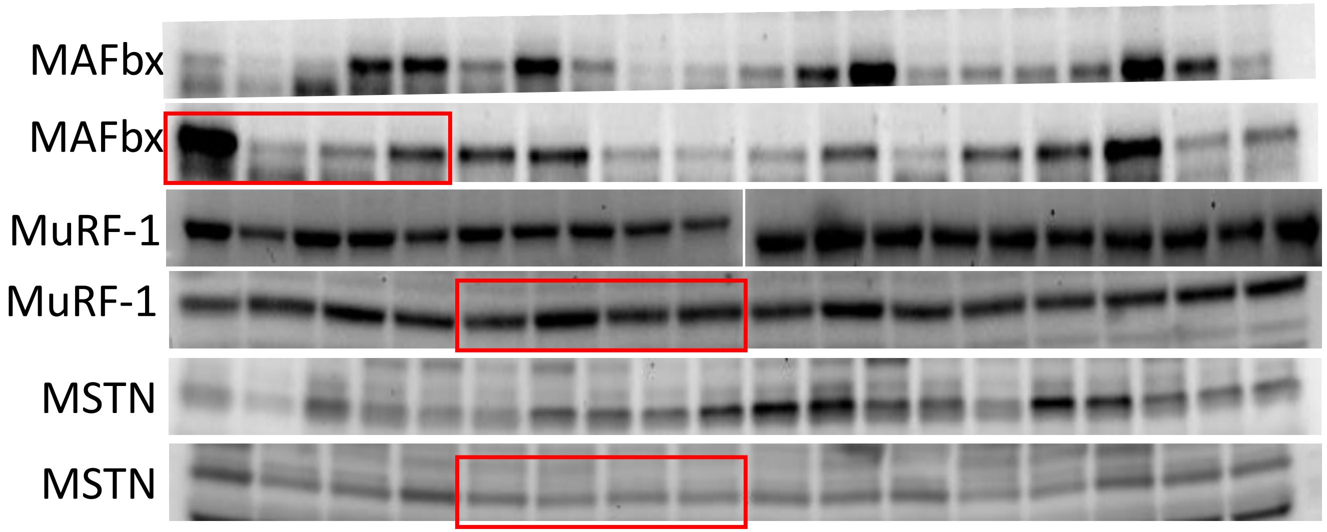

From left to right, blots are in repeats of No-stress water (control), no-stress alcohol, stress water, and stress alcohol.

Poly-Ubiquitination

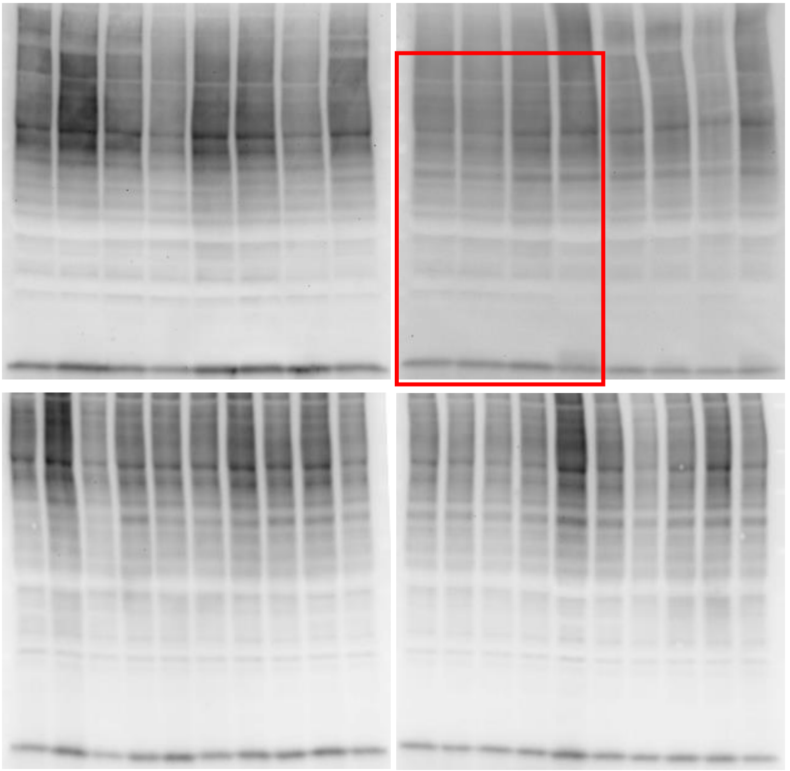

Fig 4

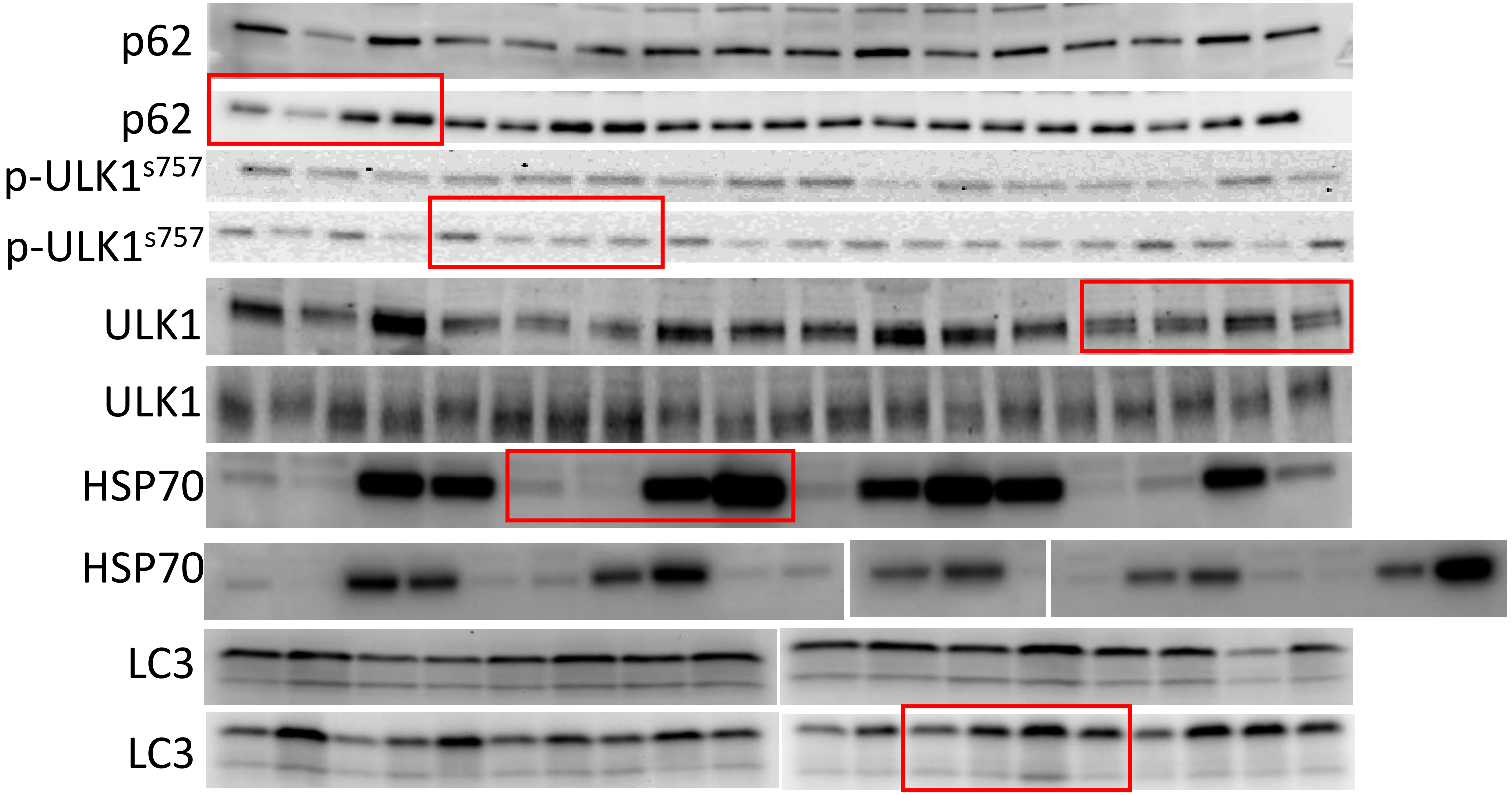

From left to right, blots are in repeats of No-stress water (control), no-stress alcohol, stress water, and stress alcohol.

Fig 5

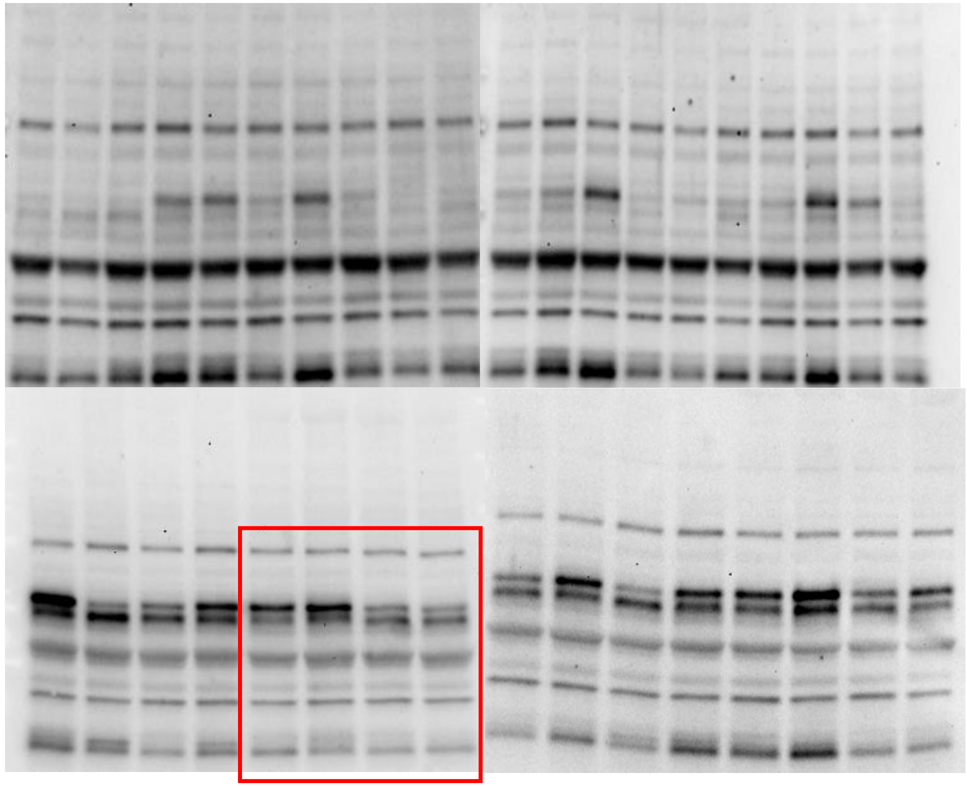

From left to right, blots are in repeats of No-stress water (control), no-stress alcohol, stress water, and stress alcohol.
